# Sensors for Wheelchair Tennis: Measuring Trunk and Shoulder Biomechanics and Upper Extremity Vibration during Backhand Stroke

**DOI:** 10.3390/s21196576

**Published:** 2021-09-30

**Authors:** Yan-Ying Ju, Wan-Ting Chu, Wann-Yun Shieh, Hsin-Yi Kathy Cheng

**Affiliations:** 1 Department of Adapted Physical Education, National Taiwan Sport University, No. 250, Wen-Hwa 1st Road, Kwei-Shan, Taoyuan 333, Taiwan; yanju@ntsu.edu.tw; 2 Department of Physical Medicine and Rehabilitation, Taipei Veterans General Hospital, Taipei 112, Taiwan; beryltipun@hotmail.com; 3 Department of Computer Science and Information Engineering, College of Engineering, Chang Gung University, No. 259, Wen-Hwa 1st Road, Kwei-Shan, Taoyuan 333, Taiwan; m0629019@cgu.edu.tw; 4 Department of Physical Medicine and Rehabilitation, Chang Gung Memorial Hospital, 5 Fu-Hsing Street, Kwei-Shan, Taoyuan 333, Taiwan; 5 Graduate Institute of Early Intervention, College of Medicine, Chang Gung University, No. 259, Wen-Hwa 1st Road, Kwei-Shan, Taoyuan 333, Taiwan

**Keywords:** wheelchair tennis, trunk, shoulder, accelerometer, biomechanics

## Abstract

This study was the first to compare the differences in trunk/shoulder kinematics and impact vibration of the upper extremity during backhand strokes in wheelchair tennis players and the able-bodied players relative to standing and sitting positions, adopting an electromagnetic system along with wearable tri-axial accelerometers upon target body segments. A total of 15 wheelchair tennis players and 15 able-bodied tennis players enrolled. Compared to players in standing positions, wheelchair players demonstrated significant larger forward trunk rotation in the pre-preparation, acceleration, and deceleration phase. Significant higher trunk angular velocity/acceleration and shoulder flexion/internal rotation angular velocity/acceleration were also found. When able-bodied players changed from standing to sitting positions, significant changes were observed in the degree of forward rotation of the trunk and shoulder external rotation. These indicated that when the functions of the lower limbs and trunk are lacking or cannot be used effectively, “biomechanical solutions” such as considerable reinforcing movements need to be made before the hitting movement. The differences between wheelchair tennis players and able-bodied players in sitting positions could represent the progress made as the wheelchair players evolve from novices to experts. Knowledge about how sport biomechanics change regarding specific disabilities can facilitate safe and inclusive participation in disability sports such as wheelchair tennis.

## 1. Introduction

Sports change individuals with disabilities in a profound way by improving their quality of life, physical health, and psycho-social wellbeing [1,2]. Although exercise for the disabled has traditionally been viewed as a recreational method for rehabilitation, athletics for the disabled has evolved to become highly competitive [3]. As global attention increased, a growing number of athletes are participating in adapted sports. One of the fastest growing wheelchair sports is wheelchair (WC) tennis [4], which has been part of the Paralympic Games since 1992. Unlike their able-bodied (AB) peers, WC tennis players typically have insufficient trunk support and lower extremity function due to spinal cord injury or amputation and compete in seated positions. However, as WC tennis has become more popular, the incidence of sport-related injuries has also increased.

WC players usually demonstrate insufficient trunk support or poor lower extremity performance. Take tennis serving as example, the sequence of muscle actions show that muscle activation begins from the proximal muscles and continues to the distal rotator cuff, suggesting that proximal trunk muscles are needed to stabilize and generate rapid movement and optimal performance [5,6]. Zattara et al. (1988) suggest that when unilateral and rapid upper limb movement is desired, muscle action starts from the calf stabilizing muscles, including the gastrocnemius and soleus muscles, and continues sequentially to the trunk [7]. It has also been reported that the ground reaction force in a tennis serve is maximized by knee joint flexion and extension. This lower extremity movement provides the main energy source, and that momentum is being transferred distally from the trunk via the shoulder during a single-handed swing through sequential momentum transfer [8,9]. Thus, the complete kinetic chain and coordination among extremity segments directly influences overall motor performance.

Limited by insufficient trunk stability and the need to use the non-dominant hand to maneuver the WC, the single-handed stroke is preferred by WC tennis players (Goosey-Tolfrey et al., 2006). Tennis elbow, the most common upper extremity injury in both WC and AB tennis [3,10,11,12], is strongly associated with the technique required for the single-handed backhand stroke [13]. For players with insufficient trunk support or lower extremity function, such as spinal cord injuries or amputations, additional upper extremity outputs are needed to compensate for the incomplete kinetic chain [14,15,16]. This compensation may result in single segment overuse, which causes high tissue burden, triggering pathological changes over time [17]. However, the compensative mechanisms of the trunk, shoulder, and wrist and how the mechanisms differ between WC and AB players have not been well investigated.

For safe, inclusive, and effective participation in disability sports, advances in research and technology play a key role in finding how disabilities specifically affect and influence the sport’s practice [2]. Combining motion capturing systems and wearable technology such as an inertial measurement unit (IMU), one can accurately track the kinematics of human movement and its acceleration/orientation patterns [18,19]. This can help to detect pathological alterations of the movement pattern [16,20,21]. Compared to able-bodied players, players with disabilities demonstrate larger individual differences, and the aforementioned method can provide objective measurement, which can be applied in real life sport. In addition to those in the general population and athletes population, the data collected can be used specifically for athletes with disabilities in athlete classifications, sport equipment customization, technique modification for injury prevention, and training protocol optimization [2].

As mentioned previously, insufficient trunk and lower extremity function add extra loads to the upper extremity during WC tennis stroke production. Additionally, WC tennis players must also adapt to the technique involved in hitting the ball while seated [22]. The shoulder is not only the essential link for transferring energy from the core to the periphery but it also functions as a hinge during frequent daily wheelchair propulsion [23]. However, to date, no study has yet investigated timing, trunk, and shoulder kinematics and impact vibration during single-handed backhand strokes, and there have been no comparisons made between AB and WC tennis players. This study aimed to examine the differences in the kinematics of trunk/shoulders and the impact vibration of the upper extremity during backhand strokes in WC tennis players and the AB players in standing and sitting positions. It was hypothesized that trunk and shoulder kinematics (swing time, acceleration, and joint angles) were significantly different among wheelchair tennis players and the able-bodied players in sitting and standing positions.

## 2. Materials and Methods

### 2.1. Participants

We enrolled 15 WC tennis players and 15 AB tennis players aged 25–60 years (Table 1). No significant difference was found between AB players and WC players in terms of height (*p* = 0.816) and weight (*p* = 0.371). Age was significantly different (*p* = 0.040). All WC players had participated in several national and international competitions. The AB players consisted of players from collegiate varsity teams. Individuals were excluded if they had any of the following conditions: (1) acute injury in the past year that restricted backhand strokes for more than two days, (2) pain in the elbows over the last two years, or (3) preferentially used double-handed strokes. All participants provided informed consents in accordance with the regulations of Chang Gung Memorial Hospital (IRB number 97-2201B).

### 2.2. Design and Procedure

The independent variable was the condition with three levels: wheelchair players (WC), able-bodied players in sitting positions (sitting), and able-bodied players in standing positions (standing). Wheelchair players were only tested in a sitting position, whereas the controls were tested in sitting and standing positions in any order.

#### 2.2.1. Performance Parameters

**Figure 1:**
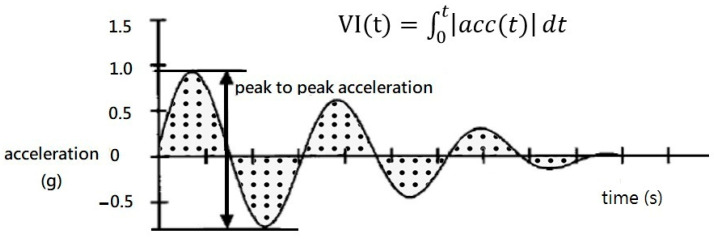
Integration of vibration Intensity over time (g*s).

The dependent variables were the backhand swing time; the trunk and shoulder kinematics including rotation/flexion/extension/abduction (shoulder only)/adduction (shoulder only) angular position/excursion; maximum velocity/acceleration of each segment; and vibration intensity (Table 2). The integrated area under the vibration curve of the maximum peak value was used to represent the vibration status. This method was based on previous tennis biomechanical studies [24,25] (Figure 1). The trunk lateral flexion was excluded because it was too limited. To describe the change in the shoulder joint flexion/extension angle, we used the following parameters: (1) the minimum and maximum shoulder joint flexion immediately before the ball impact, based on the change in the curve, as “flexion”; and (2) the maximum to minimum shoulder joint flexion after the contact point as the “extension.” In order to describe the change in the shoulder joint abduction/adduction angle, we adopted the following parameters: (1) the maximum and minimum shoulder joint abduction angle, according to the change in the curve, as “adduction”; and (2) the minimum and maximum shoulder joint abduction value after the contact point as “abduction.” In order to describe the change in shoulder rotation angle, we used the following parameters: the minimum internal rotation value before the contact point and the maximum angular value, based on the curves, as “internal rotation”; and the maximum and minimum angular value of internal rotation after the contact point as “external rotation.” The vibration intensity over time was quantified to assess the implicated energy.

#### 2.2.2. Field Preparation

The tennis courts were standard sizes according to the requirements of the International Tennis Federation, the net was set to 91.44 cm high in the middle and 106 cm at the sides, and we used a yellow tennis ball measuring 6.5 cm in diameter and weighing 57 g. A standardized KM-TN10 wheelchair (Karma Corp., Chiayi, Taiwan) was used for all participants, which had few metallic components in order to avoid potential electromagnetic artifacts. Finally, a standardized racket type (Grand Slam, Wilson Sporting Good Co., Frimley, Camberley, UK) was used for all trials, with all balls served by a Combo Pitching Machine (JUGS Sports, Tualatin, OR, USA).

**Figure 2:**
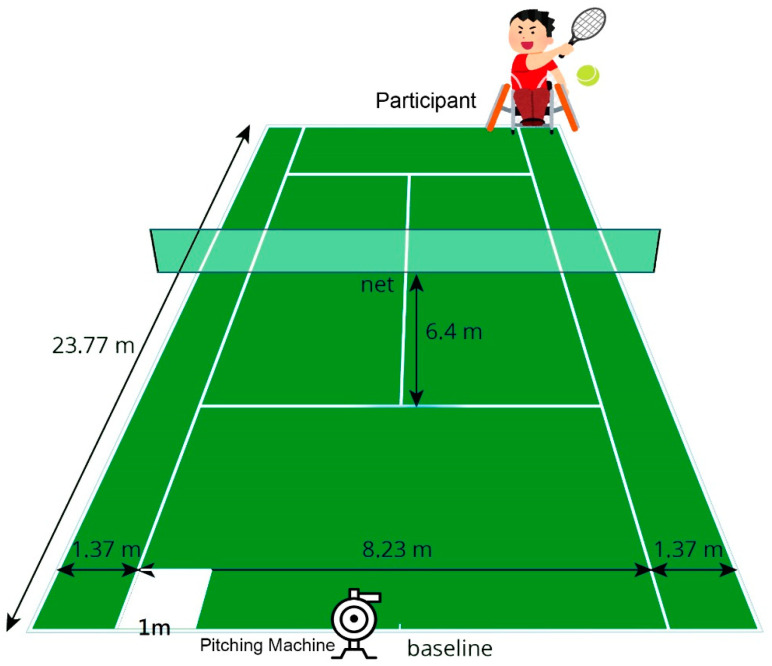
Tennis court and the target hitting area.

In the sitting position, participants sat on a WC with a seatbelt fastened at the level of the anterior inferior iliac crest, and their feet were placed on the foot rests. The racket was held by the dominant hand, with the other hand placed on the wheel for propulsion. All participants started with a 10 min warm up, including WC propelling and turning, jogging (able-bodied only), and static/dynamic stretches guided by a physical therapist. Backhand stroke practice followed the warm up procedure. A machine (Combo Pitching Machine, JUGS Sports, Tualatin, OR, USA) served the ball at a speed of 80 kph from 1 m behind the baseline of the opposite court towards the player. The player was asked to return the balls steadily and as hard as possible by using the single-handed backhand stroke with a standardized racket (Grand Slam, Wilson Sporting Good Co., Frimley, Camberley, UK) to the opposite side. Each return had to hit the 1 m^2^ target area at the corner of the baseline in order to be valid (Figure 2). The participants were asked to practice the return until they completed five valid hits.

#### 2.2.3. Motion Sensor Preparation and Data Collection

The Liberty 3D electromagnetic motion analysis system (Polhemus Corp., Colchester, Vermont, CA, USA) was used with a frame rate of 120 Hz. Prior to formal trials, the participants were prepared with sensors secured at spinal levels C7 and S1, at the acromial angle, and at 3 cm above the olecranon in accordance with the joint coordination system of the International Society of Biomechanics. Accelerometers (Crossbow, San Jose, CA, USA, 1.98 ∗ 4.45 ∗ 2.72 cm, 46 g) were secured to the racket handle (400 G), ulnar head (100 G), and lateral epicondyle of the humerus (25 G) in order to measure shock impact. All kinematic data were processed by the Motion Monitor software (Innsports Co., Chicago, IL, USA, Version 7.0) with 10 Hz (trunk), 20 Hz (shoulder and elbow), and 30 Hz (wrist) low-pass filters. The particular frequencies set for different body segments were based on the movement characteristics of those segments in order to reduce measurement noise.

**Figure 3:**
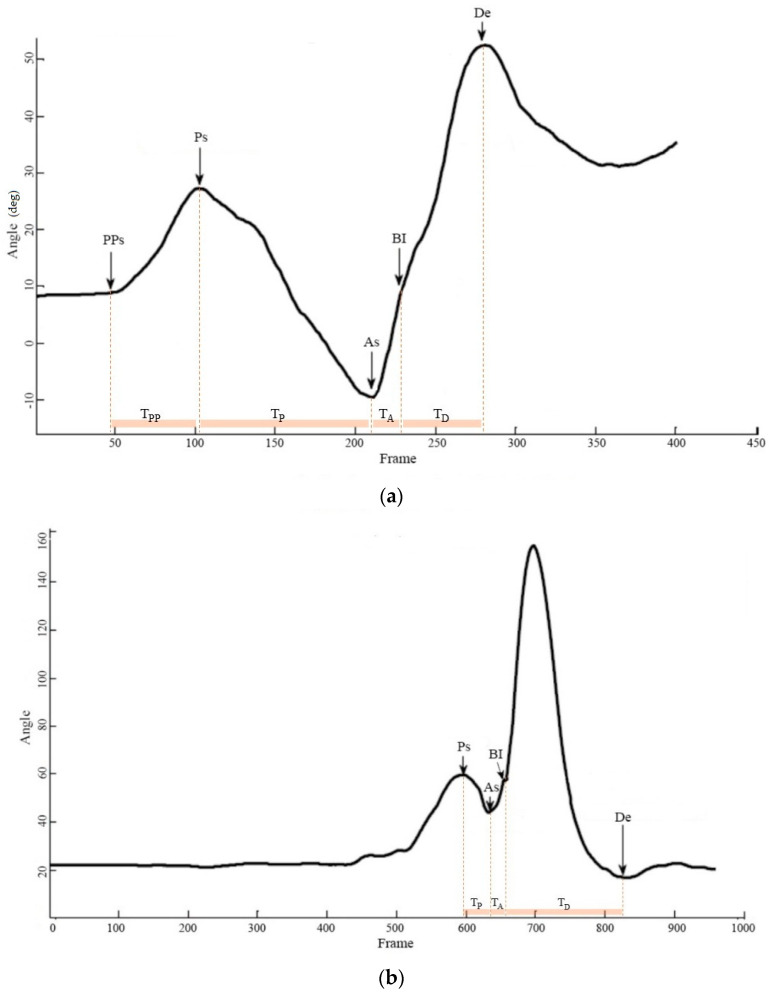
The even and phase on selected signal waveform: (**a**) trunk rotation angle in sitting condition and (**b**) shoulder flexion angle in standing condition.

The three phases of the backhand stroke/swing were preparation, acceleration, and deceleration (Giangarra et al., 1993). For trunk segment, we noticed a forward trunk rotation before the preparation phase; therefore, we denoted it as the pre-preparation phase (Table 3; Figure 3a,b). Due to the fact that stroke durations varied greatly, a normalized procedure was used. Data arrays were synchronized, and time was normalized into 100% of a stroking duration by linear interpolation, starting at the beginning of the preparation. Ball impact was set at 75% of the movement, and the deceleration phase accounts for the final 25%. The average duration for stroke completion was about 2.88 ± 1.27 s.

### 2.3. Data Analysis

Descriptive statistics were used to depict the participants’ demographics and the basic features of data. Independent *t*-tests were used to examine the differences between wheelchair and standing and between wheelchair and sitting conditions, whereas paired *t*-tests were used to compare standing and sitting conditions. All statistical analyses were performed by using the SPSS statistical software with a significance level of 0.05. Bonferroni correction was performed to adjust the significance thresholds in multiple comparisons.

## 3. Results

Angular position, angular excursion, and the relevant kinematic parameters (maximum angular velocity/acceleration and the corresponding time points) of the trunk and shoulder were compared among conditions, and the vibration intensities over time for upper extremity were also compared.

### 3.1. Backhand Swing Time

The times spent in different phases of a backhand stroke in each condition are shown in the first row grouping of Table 4. The average swing times were significantly different between the standing, sitting, and WC conditions. The standing condition took the shortest time, followed by the WC and then the sitting conditions. Although this could be attributed to the longer energy accumulation time needed in the sitting condition compared to the standing condition, it was notable that the WC condition tended to adapt and use the energy generated by fast movement to complete the swing.

In the pre-preparation phase, significantly longer time periods were found in the sitting condition than compared to the standing condition. When a sitting position was substituted for a standing position, more time was used for pre-preparation and preparation before the swinging movement. For the acceleration phase, the WC condition had significantly longer time periods than the standing condition. The sitting condition had the longest deceleration phase, followed by the WC condition, and then the standing condition, indicating that being seated in a WC means that it takes longer to complete the swinging movement. Other than the above, one can notice that the times spent in these phases varied significantly for the sitting condition, resulting in a large standard deviation.

### 3.2. Trunk Kinematics: Rotation

**Figure 4:**
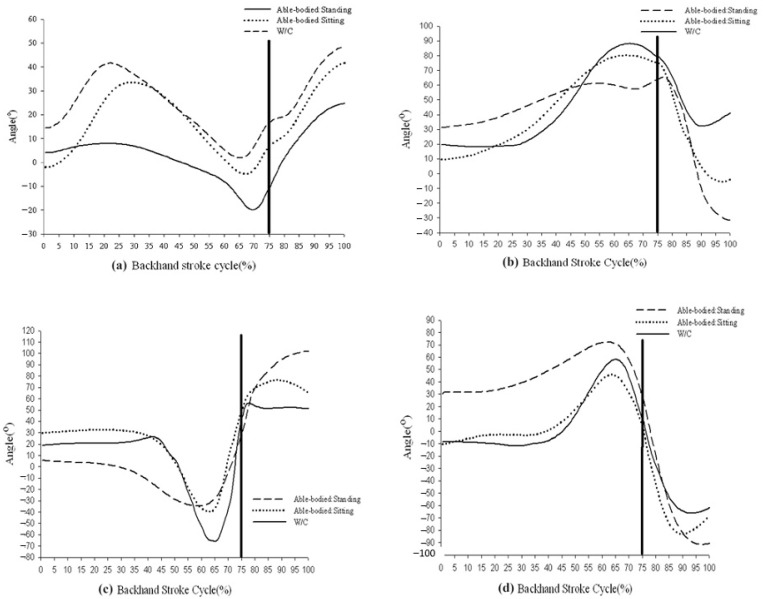
Movement excursions in three conditions: (**a**) trunk rotation in three conditions, (**b**) shoulder flexion/extension excursion in three conditions, (**c**) shoulder abduction/adduction excursion in three conditions, and (**d**) shoulder rotation excursion in three conditions.

Trunk rotation around the vertical axis was similar in the WC and sitting conditions (Figure 4a). However, forward trunk rotation was observed in the pre-preparation phase; backward trunk rotation and backward racket displacement were noted in the preparation phase; and forward trunk rotation for stroke completion and follow through were observed in the acceleration and deceleration phases. Similar movements were witnessed in the standing condition, but the angular excursions were less significant in the pre-preparation and preparation phases.

The angular position and excursions are shown for all three conditions in the second and third row grouping of Table 4. As it can be observed, there were no significant differences among the three conditions in the forward rotation excursion angle in the pre-preparation phase. The starting angular position of the three conditions differed significantly at the beginning of the preparation phase, with the backward rotation angles in the WC and sitting conditions being significantly larger than that in the standing condition. In the acceleration phase, the trunk forward rotation angle was significantly greater in the WC condition than in the standing condition. In the deceleration phase, no significant differences were noted in the forward rotation angles.

The maximum angular velocities of trunk rotation are shown in the fourth row grouping of Table 4. The velocities were significantly greater in the WC and sitting conditions than in the standing condition during forward rotation in the pre-preparation phase; however, they were significantly lower during backward rotation in the preparation phase and forward rotation in the acceleration and deceleration phases. The maximum angular accelerations for trunk rotation are shown in the fifth row grouping of Table 4. Accelerations were significantly faster for forward rotation in the WC and sitting conditions compared to the standing condition during the pre-preparation phase, but they was significantly slower during the acceleration and deceleration phases. No significant between-group differences were noted during the preparation phase. Thus, changes in kinematic parameters were similar for the WC and sitting conditions and were similarly different from those in the standing condition. Individuals in the WC condition exerted more trunk rotation compared to the standing condition before the contact point to compensate for the deficient kinetic chain and pre-accelerated to increase swing power during the acceleration phase.

### 3.3. Trunk Kinematics: Flexion/Extension

A detailed comparison of the flexion/extension kinematics is provided in Table 5, with positioning detailed in the first row grouping. The curve models for trunk angular position in the sagittal plane were such that the flexion angle of the sitting condition was maintained between 40° and 50° and was smaller than in the WC and standing conditions throughout swinging. The curve models for the WC and sitting conditions were similar, involving trunk extension before and bending after the contact point. In the standing condition, however, the trunk was bent before and extended after the contact point.

The differences in angular excursion between the three conditions are shown in the second row grouping of Table 5. In the pre-preparation phases, the trunk flexion angles were significantly different between the standing and WC conditions only, but they were significantly different between the standing condition (positive values) and the sitting and WC conditions (negative values) in the preparation phase. Trunk movement patterns were, therefore, reversed in the sitting and WC conditions in both the pre-preparation and the preparation phases. In the acceleration phase, there were no significant difference between the trunk flexion angles, but there were significant differences between the standing (negative values) and both the WC and sitting conditions (positive values) in the deceleration phase. The data suggested that the trunk was extended in the standing condition immediately before and after contact. All of the velocity and acceleration values in the WC condition were significantly greater than those in the sitting condition.

These results indicate that the range of motion for trunk extension was significantly limited in the sitting position, causing individuals in the WC and sitting conditions to use different strategies to return the ball. This involved extending the trunk just before the contact point and then quickly bending the body after the contact point. In comparison, because there were no limitations in the standing condition, the trunk was initially bent for preparation, rotated forward before and after the contact point, and finally extended.

### 3.4. Shoulder Joint Kinematics—All Movements

The results for angular excursion, maximum angular velocity, and maximum angular acceleration are shown for flexion/extension, abduction/adduction, and internal/external rotation in Table 6.

The shoulder joint models of the flexion and extension curves for the three conditions were similar. This involved shoulder joint flexion during back swinging before the contact point and extension of the shoulder joint backward when approaching the contact point (Figure 4b). However, the shoulder joint flexion curve was noticeably flatter and the shoulder joint extension curve was larger in the standing condition; thus, we used the parameters described in Materials and Methods for flexion. The shoulder joint flexion angles for angular excursion were significantly greater in the WC and sitting conditions than in the standing condition; however, although differences were present between shoulder joint extension angles, none was significant. The flexion velocity was also significantly greater in the WC condition than in the standing and sitting conditions, whereas extension velocity was highest in the standing condition, followed by the sitting condition and then the WC condition (all differences were significant). Flexion accelerations in the WC and standing conditions were also significantly faster than that in the sitting condition, but extension acceleration was significantly faster in the standing condition than in the WC condition. WC tennis players prepared for the entire movement through a larger shoulder joint flexion angle and higher angular velocity before extending the shoulder joint to return the ball.

The shoulder joint abduction/adduction curve models were similar in the three conditions, involving shoulder adduction first, followed by a large degree of shoulder abduction up to the contact point (Figure 4c). The difference was larger between the standing and sitting conditions, with both curves being flatter throughout in the standing condition; however, a distinct turning point was observed in the abduction curves for the WC and sitting conditions, so the relevant angles are reported as stated in the methods. Regarding angular excursion, the mean adduction and abduction angles in the WC condition were significantly larger than those in the standing and sitting conditions. Values for velocity and acceleration in both adduction and abduction were also significantly higher in the WC condition than those in the standing and sitting conditions, although no significant difference was observed between the latter conditions. These findings indicate that WC tennis players used large and rapid shoulder joint abduction and adduction movements, which were not used by able-bodied tennis players.

The external rotation of the shoulder joint is a key movement when returning the ball by a backhand stroke. As shown by the shoulder joint internal/external rotation curve (Figure 4d), the curve models were similar in the three conditions, with internal shoulder rotation before the contact point followed by marked shoulder external rotation when approaching the contact point. The internal rotation angle of the shoulder remained at approximately 30° in the standing condition during preparation, and the angular excursion curve was flat throughout. In order to describe the change in shoulder rotation angle, based on the curves, we used the criteria defined in Materials and Methods. For angular excursion, the mean internal rotation angle was significantly larger in the WC condition than in either the standing or sitting conditions, whereas the mean external rotation external rotation angle was significantly larger in the standing condition than in either the WC or sitting conditions. The internal rotation velocity was the highest in the WC condition, followed by the sitting condition, and it was the lowest for the standing condition (differences were significant among the three conditions). However, the external rotation velocity was significantly higher in the standing condition than in the WC or sitting. The WC condition had significantly higher acceleration during internal rotation than either the standing or sitting conditions, but there were no significant differences between the conditions in acceleration during external rotation. Thus, WC players performed a large-angle, high-velocity, and high-acceleration internal rotation of the shoulder during preparation before externally rotating it to strike the ball. The performance of external rotation was not comparable to that in the standing condition.

### 3.5. Integration of Vibration Intensity over Time

Energy was calculated as the integration of vibration acceleration over time. The integrated value of the wrist was not different among the three conditions. Rather, this value of the racket was significantly lower for the wheelchair condition than for those in the standing and sitting conditions. For elbow, the energy transmitted was significantly higher in the standing condition than that in the wheelchair and sitting conditions, with no difference between the latter two (Table 7).

As the vibration transmitted from the racket to the wrist, the energy reduction ratios (RRs) of the standing, sitting, and wheelchair conditions were 50.31%, 54.66%, and 42.86%, respectively. The ratio of the wheelchair condition was significantly lower than that of the sitting condition. When the vibration further transmitted to the elbow, the RRs of the standing, sitting, and wheelchair conditions were 83.67%, 85.63%, and 83.50%, respectively. No significant differences were found (Table 7).

## 4. Discussion

Rapid and high energy motor performances in the kinetic chain require optimal coordination among extremity segments. The lower extremities and proximal large joints are responsible for generating most of the kinetic energy and speed, while energy is efficiently transferred inter-segmentally to distal joints. Without the contribution from lower limb and torso, one can observe many compensatory movements during the backhand stroke of tennis. In summary, there were six key findings in this study. First, compared with the standing condition, the WC condition demonstrated significantly larger angular excursion, velocity, and acceleration for trunk forward rotation during the pre-preparation phase. By moving the trunk forward in the pre-preparation phase, the elastic energy built up from the backward trunk rotation in the preparation phase increases. This elastic energy can be exploited at the moment of contact between racket and ball. In proximal joints, shoulder joint flexion and internal rotation were used to improve backhand performance in the WC condition. The authors noticed that when AB tennis players changed from the standing position to the sitting position, the most significant change was observed in the degree of forward rotation of the trunk. This may be attributable to using the stretch–shortening cycle in the preparation phase to contract the right external oblique eccentrically (in a right-handed player) and to accumulate sufficient elastic potential energy to compensate for the reduced energy generated by the trunk rotation angle, thereby improving the energy of the backswing and improving swing quality.

Second, the movement patterns of trunk flexion/extension were reversed in the WC and sitting conditions compared with the standing condition. For the WC and sitting conditions, the trunk was first extended and then flexed, while for the standing condition, the trunk was first flexed and then extended. For trunk rotation in WC condition, large reversed angular excursion, velocity, and acceleration were generated by forward rotation before backward rotation in the preparation phase. Third, the WC condition demonstrated greater angular excursion, velocity, and acceleration during shoulder joint flexion when compared with the standing condition. The large angle and high angular velocities of flexion before the acceleration phase were preparatory movements for subsequent extension. However, as shown in the curve, the shoulder extension angle was limited in the WC condition. Thus, players in the WC condition were likely to increase shoulder flexion in order to compensate for insufficient shoulder extension. These compensation increases angular excursion, velocity, and acceleration of the shoulder joint and trunk, suggesting that the stretch–shortening cycle may be employed to increase motor power. This cycle involves eccentric contraction before movement to produce elastic potential energy and stimulation of sensory receptors, such as the muscle spindle, to coordinate the agonist and synergistic muscles to contract rapidly and enlist other muscle groups. The use of this strategy to output the greatest amount of energy in a short time is often observed in specific sport movements that require higher energy output, such as pitching in baseball [26,27].

Fourth, the shoulder abduction and adduction values for angular excursion, velocity, or acceleration were always significantly higher in the WC condition than in the standing condition. Elliot (2006) proposed that shoulder abduction contributes to approximately 12.9% of the ball speed and is a secondary acceleration mechanism during a tennis serve [28], and Reid et al. (2007) mentioned that WC tennis players increase their shoulder abduction angles by 15° to compensate for their lowered height on the WC during a serve [9]. Although the present study focused on the backhand stroke, we found a similar increase in shoulder abduction in the WC condition. From the perspective of sports injury, it should be noted that excessive shoulder joint abduction in a backhand swing can easily result in injury. Consistent with this, Giangarra et al. (1993) suggested that extensive shoulder joint abduction and wrist flexion during a backhand stroke could result in the accumulation of impact vibration during ball contact, causing an injury known as the leading elbow, which is prevalent in players who prefer single-handed backhand swings [29]. According to the shoulder abduction/adduction kinematics observed in this study, such movements may result in a high percentage of sport injuries such as tennis elbow in WC tennis players.

Fifth, shoulder internal rotation before the ball impact was significantly larger in the WC condition than in the standing condition, whereas the external rotation angle and angular velocity were significantly lower in the WC condition than in the standing condition. Elliot et al. proposed that, in a tennis serve, shoulder joint rotation contributes to approximately 54.2% of the ball speed and is key to acceleration. In an effective backhand swing, shoulder external rotation also plays a critical role, and the energy stored by external rotation can increase performance [28,30]. However, in a previous study of WC tennis serves by Reid et al. (2007), the external rotation angle was 20° smaller in WC tennis players compared with AB tennis players [9]. Elliot proposed that lower extremity drive in AB tennis players was closely related to shoulder joint external rotation and could effectively transfer angular momentum to the upper extremities, thereby improving external rotation of the shoulder joint [28]. Our results indicated that the external rotation angle decreases significantly when AB tennis players switch from a standing to a sitting position. We speculated that extensive shoulder joint outer rotation caused the center of gravity to tilt outwards, with a bias toward the swinging side, which compromised balance.

Finally, significantly lower racket vibration was found in the wheelchair condition. Although the level of vibration at wrist and elbow was similar across conditions, the vibration reduction ratio from racket to wrist in the wheelchair condition was remarkably lower than other reductions pairs. Compared to standing and sitting conditions, the vibration absorption function in the upper extremity of wheelchair players was not as efficient. Since vibrations generated between 15 and 700 Hz could result in tennis elbow [24], if this vibration cannot be efficiently absorbed in wheelchair players, chances of repetitive muscle injury increase. To better understand the mechanism and prevalence of tennis elbow occurrence, further investigations on electromyography and the kinetics of the related muscle are needed.

There are limitations in this study. First, the wheelchair tennis players recruited in this study had different underlying injuries and disabilities that may have had different trunk and lower extremity function. It is also possible that they were not have accustomed to the wheelchair provided to all participants. Second, the ages between AB and WC players differ significantly, and the kinematic data should be adjusted when necessary and should be interpreted with caution. Furthermore, because the change in kinematic parameters of the lower extremities was not recorded for able-bodied tennis players during the backhand swing, their lower extremity status was unknown. These issues could be remedied in a future study.

## 5. Conclusions

This is the first study to compare the differences in trunk/shoulder kinematics and impact vibration of the upper extremity during backhand strokes in wheelchair tennis players and able-bodied players in standing and sitting positions. Our results demonstrate that there are significant differences between the trunk and upper extremity movements of WC and AB tennis players, with a reinforcing preparatory movement used in WC tennis players before the striking movement. Wheelchair players do not have the inadequate power and energy contributions from their lower extremities and trunk; thus, the distal ends of the kinetic chain must generate energy through a catch-up phenomenon that can overload the upper distal joints through excessive movement. Our results offer athletes, trainers, and clinicians valid reference material about the kinematics of the backhand stroke in order to ensure that appropriate training/rehabilitation can be delivered to strengthen the trunk and shoulder in wheelchair tennis players. It is also anticipated that the findings about how body biomechanics change from regular tennis to wheelchair tennis can facilitate safe and inclusive participation in disability sport.

## Figures and Tables

**Table 1:** Participant demographics.

	Able-Bodied Players	Wheelchair Players
Gender (male/female)	15/0	14/1
Age (years)	28.6 ± 10.1	41.2 ± 7.7
Height (cm)	173.7 ± 5.7	164.5 ± 5.9
Body weight (kg)	68.5 ± 11.1	58.2 ± 9.9
Dominant arm (right/left)	15/0	14/1
Impairment		
Poliomyelitis	-	11
Lower T-level spinal cord injury	-	3
Bilateral above-knee amputation	-	1
Years of playing	7.2 ± 6.6	9.9 ± 5.8
Hours of playing per week	6.1 ± 3.2	7.3 ± 4.1

**Table 2:** Definition of the measuring parameters.

Abbreviation (Unit)	Parameter	Definition
T (s)	Backhand swing time	From the starting of pre-preparation to the ending of the deceleration phase
AP (degree)	Angular position	Location in the angular system
AE (degree)	Angular excursion	Angular displacement from one point to the next
MAV (degree/s)	Maximum angular velocity	The maximum change of position in a certain time span
MAA (degree/s^2^)	Maximum angular acceleration	The maximum change of velocity in a certain time span
VI (g*s)	Vibration intensity over time	Integration of vibration acceleration over time

**Table 3:** Definition of event and phase on the signal waveform.

Event	Definition
PPs	Starting of pre-preparation phase
Ps	Starting of preparation phase
As	Starting of acceleration phase
BI	Ball impact
De	Ending of deceleration phase
**Phase**	
T_PP_	Time spent in pre-prepareation phase
T_P_	Time spent in preparation phase
T_A_	Time spent in acceleration phase
T_D_	Time spent in deceleration phase

**Table 4:** Comparison of the time spent, trunk position, angular excursion, maximum angular velocity, and maximum angular acceleration of three different conditions.

Trunk Rotation	Standing			Sitting			WC			Between-Group Comparison		
	Mean + SD									P^1^	P^2^	P^3^
T_PP_ (s)	0.40	+	0.17	0.53	+	0.13	0.46	+	0.13	0.013 *	0.262	0.181
T_P_ (s)	0.70	+	0.23	0.75	+	0.35	0.80	+	0.13	0.569	0.185	0.634
T_A_ (s)	0.11	+	0.02	0.22	+	0.27	0.19	+	0.06	0.147	0.000 *	0.655
T_D_ (s)	0.40	+	0.10	0.65	+	0.16	0.52	+	0.10	0.000 *	0.002 *	0.011 *
AP-PP_S_ (degree)	5.5	+	4.5	0.9	+	23.8	14.5	+	18.1	0.493	0.073	0.092
AP-P_S_ (degree)	10.8	+	6.2	46.6	+	18.1	42.3	+	17.2	0.000 *	0.000 *	0.516
AP-A_S_ (degree)	−19.3	+	14.2	−0.4	+	12.9	1.2	+	10.9	0.001 *	0.000 *	0.711
AP-BI(degree)	−9.2	+	11.7	11.2	+	13.6	16.9	+	11.3	0.000 *	0.000 *	0.232
AP-D_e_ (degree)	25.7	+	8.0	48.3	+	20.5	48.5	+	15.8	0.003 *	0.000 *	0.980
AE-PP_S_ (forward rot., degree)	5.3	+	4.1	45.8	+	23.8	27.8	+	17.9	0.000 *	0.000 *	0.029
AE-P_S_ (backward rot., degree)	−30.1	+	15.5	−47.1	+	16.0	−41.1	+	11.0	0.010 *	0.032 *	0.249
AE-A_S_ (forward rot., degree)	10.0	+	4.7	11.7	+	9.6	15.7	+	6.8	0.635	0.001 *	0.203
AE-D_e_ (forward rot., degree)	34.9	+	14.4	37.1	+	11.9	31.6	+	9.9	0.821	0.467	0.186
MAV-T_PP_ (forward rot., degree)	31.7	+	16.3	212.4	+	162.9	148.5	+	118.3	0.000 *	0.000 *	0.086
MAV-TP (backward rot., degree)	−144.3	+	70.5	−101.0	+	27.7	−110.8	+	31.2	0.000 *	0.000 *	0.089
MAV-TA (forward rot., degree)	202.4	+	75.4	135.2	+	80.9	159.1	+	61.2	0.000 *	0.000 *	0.077
MAA-TPP (forward rot., degree)	426.6	+	217.3	1528.8	+	1379.2	1273.4	+	1118.9	0.000 *	0.000 *	0.672
MAA-TP (backward rot., degree)	−1308.8	+	716.9	−1801.2	+	1646.5	−1625.5	+	1113.1	0.187	0.169	0.713
MAA-TA (forward rot., degree)	3142.3	+	1302.7	1759.3	+	874.2	1837.6	+	643.8	0.000 *	0.000 *	0.547

Significance level for standing and sitting (P^1^); for standing and wheelchair conditions (P^2^); for sitting and wheelchair conditions (P^3^); *: *p* < 0.05.

**Table 5:** Comparison of trunk flexion/extension position, angular excursion, maximum angular velocity, and maximum angular acceleration of three different conditions.

Trunk Flexion/Extension	Standing			Sitting			WC			Between-Group Comparison		
	Mean + SD									P^1^	P^2^	P^3^
AP-PP_S_ (degree)	17.1	+	9.4	54.6	+	13.0	27.1	+	16.6	0.000 *	0.052	0.000 *
AP-P_S_ (degree)	19.3	+	9.8	54.4	+	12.8	25.3	+	19.2	0.000 *	0.286	0.000 *
AP-A_S_ (degree)	23.3	+	11.1	47.9	+	17.2	19.4	+	18.2	0.000 *	0.493	0.000 *
AP-BI (degree)	22.9	+	12.5	48.2	+	15.9	16.0	+	21.1	0.000 *	0.285	0.000 *
AP-D_e_ (degree)	10.9	+	13.0	49.4	+	12.4	22.0	+	24.2	0.000 *	0.127	0.001 *
AE-PP_S_ (degree)	2.2	+	2.2	−0.2	+	9.3	−1.7	+	5.6	0.392	0.017 *	0.588
AE-P_S_ (degree)	4.0	+	7.6	−6.6	+	10.2	−5.9	+	11.7	0.006 *	0.01 *	0.868
AE-A_S_ (degree)	−0.4	+	6.7	0.3	+	4.0	−3.5	+	8.8	0.839	0.291	0.153
AE-D_e_ (degree)	−12.0	+	12.0	1.3	+	6.5	6.0	+	11.8	0.003 *	0.000 *	0.195
MAV-flexion (degree)	41.7	+	22.3	−52.7	+	32.9	−74.3	+	45.3	0.000 *	0.000 *	0.000 *
MAV-extension (degree)	−731	+	43.1	55.6	+	25.3	76.2	+	54.2	0.000 *	0.000 *	0.003 *
MAA-flexion (degree)	557.6	+	311.8	−527.1	+	344.6	−787.9	+	549.4	0.000 *	0.000 *	0.000 *
MAA-extension (degree)	−1037.4	+	614.7	849.6	+	389.6	1253.5	+	714.9	0.000 *	0.000 *	0.000 *

Significance level for standing and sitting (P^1^); for standing and wheelchair conditions (P^2^); for sitting and wheelchair conditions (P^3^). *: *p* < 0.05

**Table 6:** Comparison of shoulder joint flexion/extension, abduction/adduction, internal rotation/external rotation angular excursion, maximum angular velocity, and maximum angular acceleration of three conditions.

Shoulder Joint Flexion(F)/Extension(E)	Standing			Sitting			WC			Between-Group Comparison		
				Mean + SD						P^1^	P^2^	P^3^
AE-F (degree)	39.6	+	15.2	72.1	+	13.0	58.4	+	−69.3	0.000 *	0.000 *	0.608
AE-E (degree)	−140.2	+	74.2	−119.6	+	61.2	26.1	+	44.2	0.139	0.060	0.319
MAV-F (degree)	132.6	+	62.9	181.3	+	69.5	232.9	+	80.1	0.263	0.000 *	0.003 *
MAV-E (degree)	−1125.7	+	1050.1	−867.9	+	811.0	−633.8	+	712.8	0.000 *	0.001 *	0.048 *
MAA-F (degree)	1857.2	+	1216.4	1360.9	+	628.2	2085.4	+	897.3	0.005 *	0.928	0.000 *
MAA-E (degree)	−21,749.3	+	28,506.7	−15,203.1	+	19,820.5	−11,903.2	+	15,652.4	0.213	0.007 *	0.200
**Abduction(Abd)/Adduction(Add)**												
AE-Abd (degree)	−52.4	+	22.5	−55.3	+	62.1	−137.0	+	67.8	0.792	0.000 *	0.006 *
AE-Add (degree)	131.8	+	26.7	83.0	+	74.3	182.8	+	78.5	0.057	0.001 *	0.004 *
MAV-Abd (degree/s)	−263.0	+	14.9	−362.4	+	555.0	−1719.0	+	1904.2	0.702	0.000 *	0.000 *
MAV-Add (degree/s)	938.4	+	500.2	902.5	+	1152.5	2426.2	+	2229.5	0.525	0.000 *	0.000 *
MAA-Abd (degree/s^2^)	−2544.4	+	2747.2	−6723.9	+	15,198.9	−45,600.5	+	61,857.8	0.073	0.000 *	0.000 *
MAA-Add (degree/s^2^)	16,733.6	+	21,053.8	19,612.7	+	34,336.1	65,960.4	+	70,562.7	0.768	0.000 *	0.000 *
**Internal rotation(IR)/External rotation(ER)**												
AE-IR (degree)	47.9	+	16.7	55.3	+	12.2	72.7	+	21.8	0.064	0.002 *	0.012 *
AE-ER (degree)	−170.4	+	22.1	−134.5	+	14.9	−127.9	+	27.6	0.000 *	0.000 *	0.426
MAV-IR (degree/s)	166.6	+	66.1	194.8	+	65.5	280.6	+	95.3	0.000 *	0.000 *	0.000 *
MAV-ER (degree/s)	−718.6	+	181.4	−612.6	+	157.5	−618.9	+	200.6	0.006 *	0.000 *	0.807
MAA-IR (degree/s^2^)	1575.4	+	981.8	1647.6	+	764.9	2529.6	+	1097.0	1.000	0.000 *	0.000 *
MAA-ER (degree/s^2^)	−8374.7	+	4816.5	−8247.6	+	3312.5	−8460.5	+	3587.7	0.319	0.541	0.508

Significance level for standing and sitting (P^1^); for standing and wheelchair conditions (P^2^); for sitting and wheelchair conditions (P^3^); *: *p* < 0.05.

**Table 7:** Integration of vibration intensity (VI) of the racket (400 G), wrist (100 G), and elbow (25 G) for three conditions.

		Standing		Sitting			WC	Between-Group Comparison		
		Mean + SD	RR (%)	Mean + SD	RR (%)	Mean + SD	RR (%)	P^1^	P^2^	P^3^
**VI** (g*s)	racquet	4949.69 + 1107.75		4949.52 + 116.92		3894.33 + 585.84		0.999	0.006 *	0.006 *
	wrist	2459.49 + 544.57	50.31	2243.91 + 546.17	54.66	2225.21 + 319.78	42.86	0.066	0.202	0.918
	elbow	808.45 + 214.75	83.67	711.32 + 211.16	85.63	642.58 + 227.13	83.50	0.048 *	0.038 *	0.373

RR: Reduction Ratio. Significance level for standing and sitting (P^1^); for standing and WC conditions (P^2^); for sitting and WC conditions (P^3^). *: *p* < 0.05.

## Data Availability

Data presented are available upon request from the corresponding author. The data are not publicly available due to privacy concerns.
